# Dialogue as a Response to the Psychiatrization of Society? Potentials of the Open Dialogue Approach

**DOI:** 10.3389/fsoc.2021.806437

**Published:** 2021-12-22

**Authors:** Sebastian von Peter, Tomi Bergstrøm, Irene Nenoff-Herchenbach, Mark Steven Hopfenbeck, Raffaella Pocobello, Volkmar Aderhold, Mauricio Alvarez-Monjaras, Jaakko Seikkula, Kolja Heumann

**Affiliations:** ^1^ Medical School Brandenburg, Rüdersdorf, Germany; ^2^ Department of Psychology, University of Jyväskylä, Jyväskylä, Finland; ^3^ Offener Dialog Leibzig e.V, Leibzig, Germany; ^4^ Department of Health Sciences, Norwegian University of Science and Technology, Gjøvik, Norway; ^5^ National Research Council of Italy, Institute of Cognitive Sciences and Technologies, Rome, Italy; ^6^ Department of Psychiatry, Charité University Medicine, Berlin, Germany; ^7^ Department of Clinical, Educational and Health Psychology, University College London, London, United Kingdom

**Keywords:** psychocentrism, psychiatrization, professionalism, need-adapted, dialogical, medicalization, psychologization, sanism

## Abstract

In recent decades, the use of psychosocial and psychiatric care systems has increased worldwide. A recent article proposed the concept of psychiatrization as an explanatory framework, describing multiple processes responsible for the spread of psychiatric concepts and forms of treatment. This article aims to explore the potentials of the Open Dialogue (OD) approach for engaging in less psychiatrizing forms of psychosocial support. While OD may not be an all-encompassing solution to de-psychiatrization, this paper refers to previous research showing that OD has the potential to 1) limit the use of neuroleptics, 2), reduce the incidences of mental health problems and 3) decrease the use of psychiatric services. It substantiates these potentials to de-psychiatrize psychosocial support by exploring the OD’s internal logic, its use of language, its processes of meaning-making, its notion of professionalism, its promotion of dialogue and how OD is set up structurally. The conclusion touches upon the dangers of co-optation, formalization and universalization of the OD approach and stresses the need for more societal, layperson competencies in dealing with psychosocial crises.

## Introduction

In recent decades, the use of psychosocial and psychiatric care systems has increased worldwide, even though the incidence and prevalence of so-called “mental disorders” have remained relatively stable (Beeker et al., 2021). A recent article, to which this manuscript responds, proposed the concept of psychiatrization as an explanatory framework, describing multiple processes responsible for the spread of psychiatric concepts and forms of treatment (ibid): psychiatrization can be promoted by political or psychiatric actors themselves (top-down), as well as by citizens or users (bottom-up), and can lead to various negative social effects, such as an expansion of diagnostic categories, an increasing use of psychotropic drugs or the pathologization of life challenges. Accordingly, psychiatric concepts that prevent or at least curtail the processes of psychiatrization are of particular importance.

This article aims to introduce the Open Dialogue (OD) approach and explore its potential for engaging in less psychiatrizing forms of treatment or support in psychosocial crises. OD is a multi-professional, continuous, needs- and outpatient-oriented model of psychiatric support for crisis, developed initially in Finland and then applied in more than 30 countries. In OD, the social network and the user are involved in joint treatment planning and treatment engagement from the beginning and throughout the whole therapeutic process (sometimes for years if necessary). A central element is the use of network meetings, involving the service users and their social or professional environments to enable a mutual and deeper understanding of the current crisis, as well as draw on the creativity and resources of the network and make joint decisions for further courses of action. All additional treatment elements, such as individual psychotherapy, medication, nursing, social work and others, are provided and integrated as needed. In case of hospitalisation, the same team continues to work with the individual of concern and the network as a whole.

In Finland, where OD was developed, network meetings are embedded in a specific reorganization of the entire help system, according to the following basic organizational principles (Aaltonen et al., 2011; Seikkula et al., 2011; Beeker et al., 2021) immediate help in crises, ideally within 24 h (Aaltonen et al., 2011), involvement of the social network through network meetings from the beginning of the treatment (Seikkula et al., 2011), flexibility and mobility with regards to the needs of the network in terms of frequency, location and participants in network meetings (Bergström et al., 2021), responsibility for the organization and implementation of the entire treatment process by the treatment team and (von Peter et al., 2019) psychological continuity or ensuring the continuity of relationships and common understandings over the entire course of treatment. Thus, OD as an approach depends on structural principles that enable its implementation, which may require a substantial re-shaping of the mental health care system in which it is embedded.

Existing literature describes the many benefits and positive effects of OD in client outcomes (Aaltonen et al., 2011; Seikkula et al., 2011; Bergström et al., 2021). Among others, it has been shown that its largely non-institutional and non-medicalized approach fits well with contemporary human rights perspectives which suggests that its basic network perspective promotes a contextual and relational understanding of mental well-being (von Peter et al., 2019). OD opens a space in which all participants can express themselves equally, and aims to strengthen mutual respect, autonomy and self-determination. In this respect, it seems to be a suitable model to advance an urgently needed reform of the mental health care systems worldwide (Bartlett and Schulze, 2018; WHO, 2021).

Complementary to this idea, the present paper weighs the potential of the OD approach to implement support in a less psychiatrizing way. The manuscript to which we respond (Beeker et al., 2021) mentions the possibility of de-psychiatrization, which demonstrate that psychiatrization is not a deterministic one-way road. Accordingly, we will focus in the following on the potentials of the OD approach to de-psychiatrize mental health care by either reversing psychiatrization that has already occurred or prevent it from the beginning. Thereby, we will focus on top-down processes of de-psychiatrization, first, because OD is a support service that originated in psychiatry and, thus, operates top-down by definition, and second, because we as authors all offer OD rather than receive it which also implies a top-down perspective. In terms of interaction, the described top-down effects of OD can also trigger or reduce bottom-up processes, the potential effects of which are not explored here in detail in the following due to our roles as OD practitioners and researchers.

## Effects of Open Dialogue

Since its development in Western Lapland, Open Dialogue has been studied in five cohort studies with first-episode psychotic disorders (Lehtinen et al., 2000; Seikkula et al., 2006; Seikkula et al., 2011; Bergstrøm et al., 2017), and currently a large cluster-randomized control trial (ODDESSI trial) is being conducted in the United Kingdom. These cohort studies show promising results even regarding OD’s potential for de-psychiatrization, including a significant reduction of inpatient stays (i.e. hospital days and re-admissions) as well as lower relapse rates over time in all cohorts (Seikkula et al., 2006; Seikkula et al., 2011; Bergstrøm et al., 2018). In addition, the results show a re-integration into work and education of up to 84% of the participants and a considerably low and infrequent use of neuroleptic medication both initially (28–50%) and during the course of intervention (11–29%) (Seikkula et al., 2006; Bergstrøm et al., 2018). In the comparison between the individual cohorts, shorter and less severe psychotic episodes were described as well as a dramatic reduction (up to 82%) of clients with residual symptomatology. Additionally, a decrease in the use of psychiatric services and frequency of network meetings were reported (Seikkula et al., 2006; Aaltonen et al., 2011; Bergstrøm et al., 2017), and significantly fewer disability allowances were used compared with historical control groups (Seikkula et al., 2006; Bergstrøm et al., 2018). Overall, across all cohorts from 1992 to 2005, evidence showed that the treatment outcomes achieved in each case remained fairly stable over the entire period or even increased over time (Bergstrøm et al., 2018).

The results of the cohort studies in Western Lapland paint an alternative picture to that of traditional treatment for psychotic crises which relies heavily on drug treatment and is associated with high socioeconomic costs (Charlson et al., 2018; He et al., 2020). Moreover, the described effects of OD are an indication that this form of support has the potential to counteract and potentially prevent further expansion of psychiatric concepts and psychiatrized treatment services at several levels (Beeker et al., 2021): a limited use of neuroleptics (Aaltonen et al., 2011), reduced incidences of mental illness and a more restrained use of diagnostic categories and (Seikkula et al., 2011) an overall decrease in the use of psychiatric care services. Yet, it should be noted that these outcomes could only be achieved through the comprehensive structural changes in the participating Finnish catchment areas. The extent to which OD has a similar potential for de-psychiatrization without these structural changes remains unclear. Accordingly, the question arises in which way or through which therapeutic elements the de-psychiatrizing potentials of OD are mediated? In the following section, we investigate five potentially decisive aspects (Beeker et al., 2021): the use of language (Aaltonen et al., 2011), the processes of meaning-making (Seikkula et al., 2011), the notion of professionalism (Bergström et al., 2021), the promotion of dialogue and (von Peter et al., 2019) some structural aspects of OD.

## Use of Language

Apart from treatment planning, a primary goal of network meetings in OD is to foster a shared, polyphonic (i.e. eliciting multiple perspectives) dialogue among participants by using a specific form of language (Seikkula et al., 2001; Seikkula and Trimble, 2005). As described in its principles (Olson et al., 2014), support within the framework of OD relies on the use of everyday terms and non-psychiatric language or terminology. Instead of primarily following a certain agenda (e.g. by asking diagnostic questions), the network meeting facilitators pay attention to the words and stories of the network participants, notice expressions and themes they perceive as important to the network and use them to further expand on ideas (e.g. by repeating individual utterances without paraphrasing or interpreting). Allowing for long silences and being curious about key words that seem significant, is also common. In this way, key words can become central subjective concepts for the communication between the network participants and action-guiding terms useful for planning further steps for support. In doing so, ambiguity is explicitly encouraged and valorized: different meanings and explanatory models of psychosocial crises are allowed to coexist and are essential for understanding and establishing relationships among network participants.

Instead of using a medicalizing language or psychiatric concepts and classifications, OD support focuses on elaborating individual meanings, bringing idiosyncratic narratives into exchange, and using contextual language anchored in everyday life. Behaviors and interactions are not explained by diagnoses or classificatory concepts (unless raised by members of the network) but are understood by contextualizing them as adaptations to stressful life situations or the life histories of individuals or the network. This way, a deeper understanding of the participants among themselves is made possible and solutions can be found collaboratively. Each network participant is supported in this process to contribute their own perspective and find their own terms and concepts. In this context, psychiatric or psychological explanatory approaches are usually dispensed with altogether or are provided–at most–as *one* perspective among many for understanding and explanation, while ideas by the clinical team are held lightly and offered tentatively.

Breaking the interpretive sovereignty of psychiatric language is an integral part of OD, which may explain a significant part of its de-psychiatrizing potential. Instead of using the often stigmatizing psychiatric language and concepts (Rose and Thornicroft, 2010) or using standardized treatments tailored for specific presentations rather than to the persons of concern, the participating networks gain a unique expertise about the explanation and/or solution to their own life situation. From a de-psychiatrizing perspective, the individual language is preserved as a tool for understanding and dealing with crises which can, in the long run, have the potential to de-psychiatrize as it fosters idioms that are grounded in the network’s everyday life. In that sense, a bottom-up effect can also be assumed, resulting from the OD’s cultivation of a diversity of language in relation to crises, fitting the multi-layered realities of those involved and thereby offering spaces for self-empowerment in dealing with them—a hypothesis that has been supported by recent research (Bergstøm et al., 2019).

## Processes of Meaning-Making

OD evolved from the Finnish Need-Adapted Treatment approach, developed from the 1960s to the 1980s in Turku as part of the Finnish national schizophrenia project for first-time psychotically affected people over five phases (Alanen, 2009). This approach was developed as an integrative treatment model based on family therapy, network therapy and psychoanalytic concepts. This led to practices in which the participants are asked to find (new) meanings for the present crisis together during the network meetings. Crises are understood contextually as “natural” responses to challenging life events rather than explained by psychopathology or neurobiological correlates (Seikkula, 2019). They are always seen as meaningful and understandable in the context of an individual life if one only listens closely or asks carefully, thus being normalized as learned responses to a stressful situation.

Thus, during a network meeting the team listens for the meaningful and “logical” aspects of each person’s response. The participants are supported to find meaningful explanations instead of framing or understanding a behavior as “wrong” or “crazy”. In the form of a “conversational back-and-forth exchange” (Olson et al., 2014) a subtle process of understanding and responding takes place between the network participants and the team, from which meaningful stories gradually emerge that aim at grasping the frequently unspeakable dilemmas and experiences that are at the root of a given symptom. Thereby, during a phase of acute crisis, finding and discussing a single keyword may be more important than a complete story of explanation. This single word may be explored together to arrive at a shared understanding of the crisis at hand, making it more understandable to foster new possibilities to act and think for everyone.

From a methodological perspective, OD practitioners work as a team to support the process of generating and sharing meaning in two different ways related to outer and inner polyphony during the process of the network meeting (Haarakangas, 1997; Seikkula, 2008). Outer or horizontal polyphony happens when the practitioners assist the emergence of the different points of view of the members in the network by providing an opportunity for each participant to express themselves, paying attention to both what is said and to non-verbal expressions. The inner polyphony, also described as vertical polyphony, refers instead to the awareness and use of the different inner voices of both the practitioners and network participants during the network meeting. In this respect, OD meetings can be conceived places for sharing and co-producing knowledge, meanings, experiences and feelings where both professional and lay perspectives are valorized, thus leaving sufficient space for processes of de-psychiatrization.

Further, at times one or more network meeting participants may try to understand the crisis as resulting from a biological or medical problem or with the help of psychiatric nosology. These persons may react with disappointment when the dialogical engagement within the meeting also generates other explanations or attempts at meaning-making. These can be challenging moments in which psychiatric knowledge is needed as well as a profound sensitivity in order to more deeply understand the questions, thoughts and feelings that may lay behind this desire to understand a crisis in medical terms. Quite often, this understanding is simply the result of previous contact with the psychosocial system in which these types of explanations were given along the way. In this sense, OD can also be understood as a possibility to revise these bottom-up psychiatrization processes or at least question them and make them a topic for further exploration.

## Notion of Professionalism

It is obvious that this use of a non-psychiatric language, the promotion of dialogue and the associated (dialogic) attitude have profound implications for the role of those working in OD, including an impact on professional identity. This is especially true for psychiatrists who need to consider what kind of expertise, what competencies and what bodies of knowledge are needed for good implementation of a network meeting, topics that are the subject of recurrent discussion in the OD community (Holmesland et al., 2010; Borchers, 2014; Valtanen, 2019; Schubert et al., 2021). What is clear, however, is that the central expertise lies not in the transmission of knowledge by mental health workers but in their capacity to promote dialogue and the equal exchange of perspectives (Seikkula et al., 2001; Olson et al., 2014). Any treatment mandates or problem definitions do not come unidirectionally from the mental health professionals but primarily emerge from the dialogue among the network meeting participants. The network members should be allowed to decide the content of the exchange, the focus and frequency of the support and whether support is needed or not. On the other hand, the practitioners may offer tentative advice about these decisions, but their primary responsibility is to facilitate and moderate the dialogic processes. They provide the flexibility and mobility necessary to respond to the needs of the network with sufficient staff continuity throughout the treatment process.

When the network meeting practitioners contribute to the exchange, they often do so from a reflexive and personal perspective, drawing on their own private and professional experiences as needed. They certainly may also contribute with professional knowledge but primarily when this is requested by the network and then marked as only one of many possible perspectives. Furthermore, a large part of one’s own contributions is offered in the form of an explicit reflective talk between professionals in the presence of the whole network about their experience of witnessing the network process (“reflecting team”) (Andersen, 2007; Schriver et al., 2019). This kind of reflection, as a way of sharing professional expertise (Jacobsen et al., 2021), can be rejected by the network much more easily than a seemingly scientific or medicalized explanation that is often introduced with a more *de facto* stance. Thus, the practitioners contribute with their own thoughts, professional knowledge and life or work experiences in a questioning manner rather than dominating the network discussion with medical terminologies. From this point of view, the knowledge and expertise about the network are in the network itself, whereas the practitioners contribute by enabling a dialogic exchange.

This approach requires the practitioners to assume a position of “not knowing”, assuming that each person involved in the network has their own view of the situation, which may not even be comprehensible at first (Anderson et al., 1992). A person’s experience and understanding of a situation is not self-explanatory and must therefore be openly inquired about and exchanged in the network. Even if different perspectives have been shared in a network, one can never be sure whether a point of view or a problem has really been fully understood, grasped or recognized. Accordingly, hasty solutions or decisions are also to be avoided. Especially in crises, this is in stark contrast to the usual, risk-averse, security-seeking processes of psychiatric care. A central principle in the implementation of network talks is, therefore, a tolerance for uncertainty: while the facilitators have an inner openness for the processes described, they provide a framework that enables exchange and creating the opportunity for previously unheard ideas and explanations for the crises to be heard (Olson et al., 2014).

Thereby, a transparent and open way of communication (i.e. making their thoughts and feelings open to the network) is another principle of practicing OD. Since traumatic experiences and experiences of powerlessness are of great importance for developing psychosocial crises, this transparency on behalf of the practitioners can foster a sense of safety and security (Seikkula and Trimble, 2005; Seikkula et al., 2006). As such, professionalism in OD requires staff to be genuine, openly sharing fears, hopes and anxieties. Instead of “standing above” or distanced from a crisis-situation, they find themselves in the middle of it both metaphorically and concretely. As the network meetings often take place in private homes, OD practitioners are guests, adapting to the context and providing contextual responsiveness to the crisis.

Thus, the OD approach requires a strong redefinition of what counts as professionalism. In network meetings, the professionals primarily participate as human beings with feelings and emotions, who are fallible and justly uncertain about the complex, context-dependent and interactional nature of crises. As much as they may contribute with knowledge that they have accumulated through education or life and work experiences, the concrete solutions, explanations or answers must be given by the network participants themselves. From a de-psychiatrization perspective, one could argue that OD re-signifies the image of psychiatric professionals: instead of being mainly authoritative experts on the nature and management of disorders or diseases, they are now seen as specialists in facilitating dialogical conversations or interactions that also may be helpful to prevent or counteract the psychiatrization of other areas of life.

## Promoting Dialogue

In addition to the use of language described above, further practices are used in OD to promote dialogic exchanges: reflection among the professionals on the content or the structure of a network meeting, relational questions or making sure that all participants have their equal say. Network meetings are always facilitated in team, making open reflections and exchanges (i.e. in the presence of the network) between practitioners possible. Practitioners understand themselves as a part of the dialogue, paying special attention to the actualization of the present encounter. What happens in the here and now of a network meeting is often more important than the details of a long case history or extensive descriptions of symptoms. As a result, this focus on dialogical engagement during a network meeting may allow for new meanings or explanations to emerge, resulting from the actual interactions and discussions between the network members and having the potential to find relational and context-bound instead of psychiatrized solutions.

Further, OD differs significantly from traditional psychiatric practices in its active involvement of the wider network of the persons of concern. Before the first meeting (and every session thereafter), clients are asked who they think is influential for or during a crisis and, thus, should be involved in a network meeting. Network members may be family members, friends, or even contacts in authorities, employers and other persons of support. Involving people from various backgrounds and life contexts fosters a rich exchange with multiple forms of knowledge and ways of being. Experiences of violence, power relations, inequality, exclusion, isolation are example topics that are frequently discussed and point to the social but also societal, micro-political (rather than only medical) focus of this approach. Thus, crises are no longer relegated to explicitly designated and segregated spaces. The boundaries of psychiatric action are less fixed and are not tied to specific institutions or limited to a small, medical or restricted professional framework. Instead, OD support in psychosocial crises means bringing different worlds together and into exchange, resulting in a changed reality for those involved. In this way, OD shifts the focus of crisis support away from external experts towards joint dialogues with multiple actors, reducing the risk that the psychiatric assessment is removed from the reality of the people concerned. Promoting dialogue could thereby prevent top-down processes of psychiatrization in the field of psychiatric assessment.

## Structural Aspects of Open Dialogue

Structural aspects refer to how OD is implemented in daily practice and within the wider mental health care landscape. OD is not a manualized psychotherapeutic or medical intervention. However, it does follow a set of principles, identified for the purpose of training, research and implementation, that are put into use or recombined in various ways depending on the needs of the network (Olson et al., 2014). Openness is notwithstanding a central component of its implementation. OD is genuinely need-adapted, which is per definition at odds with top-down, psychiatrizing approaches commonly used in contemporary psychiatric care institutions.

Further, as mentioned above the implementation of OD in Finland was accompanied by a fundamental restructuring of the local health care structures. This involved a major reduction of hospital beds and inpatient facilities and prioritization of outpatient and outreach treatments (Seikkula et al., 2001). As a consequence, meetings were implemented in the living environment of the network: their homes or at school or work, if desired. In this respect, OD shares structural similarities to various approaches of integrated care, such as the work of FACT or ACT teams, many of which have a strong evidence base (Gühne et al., 2018).

Another important goal was developing an alternative form of support in case of psychotic crises able to reduce or dispense of a primarily psychopharmacological approach in psychiatry: A key outcome parameter was the extent to which OD helped to either eliminate the need for neuroleptic medication or to reduce it. This focus alone demonstrates how seriously OD has tried to find non-medicalizing responses to and ways of dealing with psychosocial crises. In that sense, OD can be seen as a tool for the de-medicalization of psychiatric services, a goal that seems to be central to de-psychiatrization (Beeker et al., 2021).

With network meetings at the center of support and having a contextual understanding of crises, OD can be understood as a systemic form of therapy. Psychosocial crises, the responsibility for their emergence and the way(s) out of them are distributed upon several shoulders. Thus, OD breaks with the deeply individualizing infrastructure of traditional psychiatric approaches which allow for psychiatrization processes to further expand. In fact, it may be understood as a means to de-pathologize an individual and instead contextualizing a crisis by creating an embedded understanding against the background of a wider social network. Thus, OD aims to return the responsibility for understanding, managing or overcoming crises to more than one person and, understood somewhat more broadly, to society itself. All people in the social network should be asked and feel empowered to communicate and work together to find solutions to extraordinary situations, whereas common psychiatric approaches tend to allocate this responsibility primarily to one person, the individual of concern.

At the same time, whether these structural aspects come into play depends heavily on the nature of the care context. In many countries, some of the principles mentioned can only be implemented to a limited extent because the mental care systems are highly fragmented and geared to the support of individuals, therefore hardly providing for any opportunities for continuous, systemic support work. Yet, if the OD is implemented in its full (Finnish) form, the structural aspects mentioned could contribute to its de-psychiatrizing potential.

## Advocatus Diaboli

It is important for us not to present the OD as an all-encompassing solution to de-psychiatrize support in psychosocial crises. Certainly, dialogue may not be the primary solution to all problems at hand. Further, the OD is not free of some psychiatrizing effects and probably cannot entirely be. After all, it has been developed gradually, over many years, and primarily by psychiatric or mental health professionals. Thus, even though OD has undoubtedly been based on different and *trans*-disciplinarily anchored concepts and theories over the course of years it originates in psychiatric discourses and practices and cannot be separated from them in its origins, orientation and concepts.

To give a more concrete example, the psychiatrizing risk of OD may be transmitted by its outreach approach: Despite the undisputed positive effects of outreach forms of treatment, especially in comparison to classical inpatient care (Gühne et al., 2018), moving psychosocial support into the living environment of users is, first of all, a formally psychiatrizing process. When a psychiatric concept is brought into someone’s home, it can potentially reach more people in their private living environment and thus shape the role of psychiatry in everyday life, quite independently of the type, orientation or quality of the support offered.

Secondly and argued from a somewhat broader perspective, OD is also based on the basic assumption, common at least in Western and individualistic countries, that the care of psychosocial crises requires an institutional response. Instead of dealing with these crises collectively within society, dealing with them has been delegated to staff members of an institution who are paid for it, trained for it, and consequently, always bring a limited range of response options.

Thirdly, OD both in its original application in Finland and in most cases at present is implemented within medical-psychiatric frameworks, i.e. within mental health care systems. This context of application powerfully shapes the way OD is implemented (Von Peter et al., n d). Legal regulations on professional recognition and prerequisites for care, possibilities of billing or recognition of work performed or the concrete organizational conditions of a care system are just a few examples of the many ways in which the concrete design of a health care system can influence the implementation of any mental health approach.

Fourthly, most of the staff of the institutions that currently offer OD internationally largely belong to psychiatric professions. They are mostly conventionally socialized in psychiatric or psychosocial institutions and receive OD training often only in a later stage of their career. In this respect, at least the development of Peer-Supported Open Dialogue (POD) is a promising development (Razzaque and Stockmann, 2016), which could promote the democratic and non-hierarchical orientation of OD (Bellingham et al., 2018). At the same time, the very inclusion of peer experts by experience in existing psychiatric services repeatedly raises questions about appropriation and alignment with psychiatric treatment routines and roles which are also closely related to the question of the psychiatrizing potential of OD.

These examples make it clear that the OD approach cannot be free from psychiatrizing effects either. Psychiatrization as a concept describes a development of society as a whole and is already strongly advanced in many Western societies and accordingly effective. OD is in most cases trained and applied within the existing mental health care systems, which limits its possibilities to respond to psychosocial crises in a de-psychiatrizing way, sometimes drastically, depending on the context. While adopted primarily within public mental health services (Pocobello et al., 2021), in some contexts OD is also implemented by independent associations such as “Offener Dialog e.V.” in Leipzig, where it is offered on a voluntary basis and outside the logic of psychiatric care (i.e., without the need for a diagnosis, an obligation to document or prove the fulfilment of a medical treatment mandate and without the use of psychotropic drugs). However, such projects will remain an exception or could potentially sink before setting sail without an adequate funding base.

## Conclusion

The goal of this paper was to weigh the de-psychiatrizing potential of supporting psychosocial crises with the OD approach. Although this need-adapted approach has its origins in psychiatric discourses and practices and is implemented in this setting in the majority of cases, OD holds some potential for de-psychiatrization. As shown, this potential has been demonstrated by the outcomes of the mentioned cohort studies. Further, it can be theoretically explained by the logic of the OD approach, in the way language is dealt with, the role of the staff within the network work and how this treatment approach is set up both structurally within the mental health care system and in its everyday application.

It must be said that the way OD is offered can differ significantly from the way users experience it. As described initially, we can only provide a top-down perspective due to our role as mental health professionals, OD practitioners and researchers. Thus, it is hard for us to wage whether OD can also bring about societal de-psychiatrizing change processes. This could be a subject for further investigation, as well as transdisciplinary research that critically examines, whether our more conceptual analysis of OD’s de-psychiatrizing potentials holds up in practice. Empirical data on both a public health and local level are needed to confirm that the OD approach leads to treating mental suffering in a less psychiatrizing way than other treatment approaches.

In addition, the question arises if the described effects of the OD approach in Finland may require a temporal contextualization since at the time of these cohorts the psychiatrization of society was not so advanced compared to the present. Within the last few decades, psychiatry has increasingly adopted a reductionist neurobiological model (Bracken et al., 2012) which has also changed related disciplines, such as psychology, social work, etc., as much as society as a whole. So, can the de-psychiatrizing outcomes of OD be reproduced at present, despite this seemingly un-reversible process?

In this context, we currently see the danger that OD will be appropriated to serve as a pretty cloak to cover a medical-psychiatrizing treatment (and societal) system. This danger is even more pertinent, as calls for more democratic, human rights-based, empowering or recovery-oriented psychosocial support systems seem to expand, yet often without the willingness or sufficient reflexivity to change customary routines (Von Peter and Zinkler, 2021). In this context, the OD’s primary principle of openness rather invites to fill and occupy the approach with own contents and ideologies, including medical concepts and procedures. Thus, whether the OD can have a de-psychiatrizing effect or not is not self-evident but depends on how it is implemented and whether the necessary context of care exists.

There have been and still are extensive discussions within the OD community about the extent to which OD should be formalized, also to be able to demonstrate and investigate its implementation fidelity. While some studies report resistance against standardization and replicable criteria for training and evaluation (Alvarez Monjaras, 2019; Florence et al., 2020; Hopper et al., 2020), different scales have been developed to assess organizational fidelity and clinical adherence (Olson et al., 2014; Alvarez-Monjaras et al., 2021; Lotmore et al., 2021). These scales operationalize the essential aspects of OD well and is being used in connection with the above-mentioned trial in England and upcoming studies. Yet, a more detailed description of the OD has also the disadvantage of limiting its need-adapted openness in implementation with all the dangers of its interventionist use, potentially too firmly prescribing which solutions are (or should be) found in which way for which problems.

Finally, OD is not a panacea. As stated, dialogue may not be the primary solution to all forms of mental health crisis. In an interview, psychiatrist Sandy Steingard pointed out the dangers of idealizing OD (Steingard, 2020), propagating it as the silver bullet for any psychosocial problem. OD is also man-made, error-prone and does not fit all situations of crisis. An overly dogmatic promotion does not do justice to these circumstances, and can lead to false hopes or expectations being raised among users, relatives, staff and other stakeholders. International cohort studies will try to clarify when, under which conditions and in which ways does the application of OD make sense. Indeed, OD can be considered as only one component to bring about the urgently needed changes in the mental health care system.
